# High-resolution ultrasound and magnetic resonance imaging of ulnar nerve neuropathy in the distal Guyon tunnel

**DOI:** 10.1186/s13244-023-01545-z

**Published:** 2023-11-28

**Authors:** Riccardo Picasso, Federico Zaottini, Federico Pistoia, Marta Macciò, Gabriele Rossi, Corrado Cabona, Luana Benedetti, Carlo Martinoli

**Affiliations:** 1 IRCCS Ospedale Policlinico San Martino, Largo Rosanna Benzi 10, Genoa, Italy; 2 Department of Health Sciences (DISSAL), Radiology Section, University of Genova, Via Pastore 1, Genoa, Italy

**Keywords:** High-resolution ultrasound, Peripheral neuropathy, Hand imaging, Ulnar nerve

## Abstract

**Objective:**

The aim of the present study is to describe the ultrasound (US) and magnetic resonance imaging (MRI) findings in patients with neuropathies affecting the deep (DB) and superficial (SB) branches of the Ulnar nerve (UN) and to investigate the potential role of imaging modalities in the diagnostic workup of these conditions.

**Materials and methods:**

We screened our institutional imaging database to identify patients with a diagnosis of UN mononeuropathy, and among them, we reviewed the cases where US disclosed pathological findings affecting the UN terminal divisions. In this latter subgroup, we retrieved available data on MRI and electrodiagnostic tests performed by the patients during the diagnostic workup. All the patients were evaluated with US machines equipped with 17–5-MHz, 18–4-MHz, 24–8-MHz, or 22–8-MHz probes. MRI exams were performed on a 3-T unit equipped with a 64-channel head RF coil.

**Results:**

Among 166 patients with UN mononeuropathy, we retrieved 15 patients (9%) for which US detected pathological findings affecting the UN terminal divisions, consisting of 7 cases of DB neuropathy, 4 cases of SB neuropathy, and 4 cases of combined neuropathy involving both nerves. Seven (46.7%) patients were submitted to MRI to integrate US findings. Among patients with SB and DB neuropathies, imaging allowed the identification of 7 traumatic nerve injuries, 2 nerve tumors, and 6 entrapment neuropathies, including 4 cases of nerve compression by a ganglion cyst.

**Conclusion:**

High-resolution US and MRI are accurate modalities for the investigation of patients with SB/DB neuropathy, can provide critical information on the cause of nerve damage, and guide therapeutic decisions.

**Critical relevance statement:**

High-resolution US and MRI are accurate modalities for the investigation of patients with superficial/deep branch of the ulnar nerve neuropathy. In the proper setting, US may be regarded as a first-line approach in patients with suspected neuropathies affecting these small branches.

**Key points:**

• Neuropathies affecting the distal ulnar nerve often require multimodal investigations.

• US and MRI can provide detailed morphological information about the terminal branches of the ulnar nerve.

• US may be considered as a first-line approach in suspected distal ulnar nerve neuropathies.

**Graphical Abstract:**

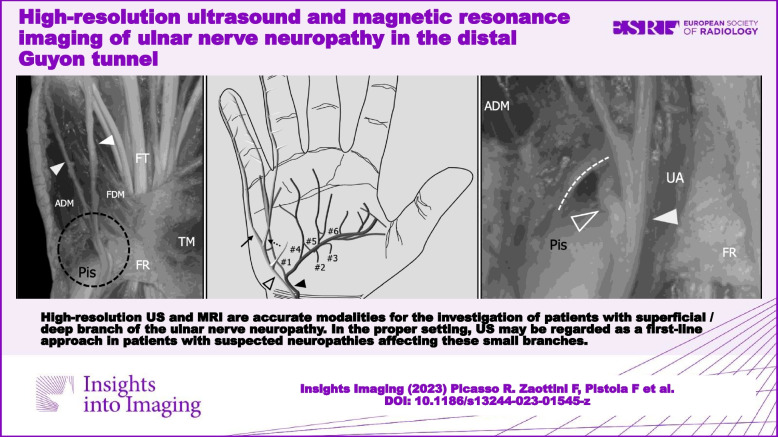

**Supplementary Information:**

The online version contains supplementary material available at 10.1186/s13244-023-01545-z.

## Background

Ulnar nerve (UN) mononeuropathy consists of a mix of neuropathic pain and paresthesia involving the last finger and a half and motor impairment in performing fine movements of the hand. Even if their prevalence among patients with UN neuropathy has not been documented, atypical cases with selective involvement of the motor or the sensory function may be encountered and represent a clinical challenge due to the unusual presentation and broad differential diagnosis, which includes central nervous system disorders, cervical roots compression, plexopathies, dysimmune diseases, and distal neuropathies of the superficial (SB) and deep (DB) branches of the UN [1].

Regarding the latter, the terminal divisions of the UN can be affected by several pathologic conditions, including traumatic injuries, fibrous bands, tumors, and ganglia [2, 3]. Indeed, whereas the DB is subjected to impingement as it engages tight fibrous tunnels, pierces thin muscle bellies, and runs in close contiguity with bones and tendons across a biomechanically exposed area, the SB is vulnerable to traumatic injuries along its long and unprotected path in the subcutaneous tissue of the palm and the fingers. The coexistence of multiple zones of possible nerve impingement in a relatively small area, the necessity of obtaining a precise indication of the level and the cause of nerve injury to minimize the extension of surgical incisions, and the scarce reliability of electrodiagnostic tests in characterizing these conditions, make imaging modalities particularly relevant in the diagnostic work-up of patients with suspected SB/DB neuropathies. A thorough knowledge of regional anatomy, a high level of expertise in nerve imaging, and the availability of modern high-end equipment represent essential prerequisites for performing a state-of-the-art radiological evaluation of the SB and the DB.

The aim of the present study is to describe the US and MRI findings in patients with SB/DB neuropathies and to investigate the potential role of imaging modalities in the diagnostic work-up of these conditions.

### Anatomical notes

**Fig. 1:**
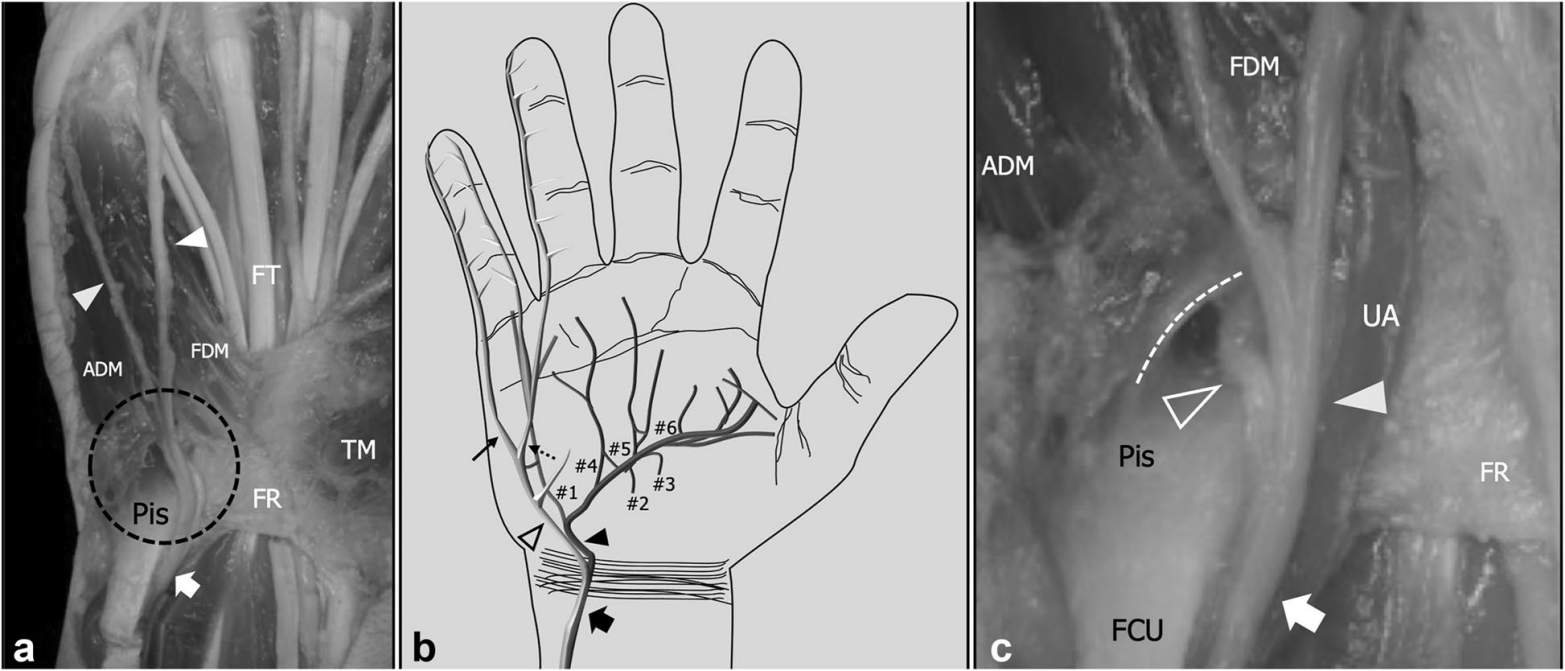
Guyon Tunnel, regional anatomy. Cadaveric specimens (**a**, **c**) demonstrate the ulnar nerve (arrow) as it engages the Guyon tunnel running on the radial side of the pisiform (Pis). Distal to the pisiform the deep branch (outlined arrowhead) crosses the pisohamate hiatus (dashed line), which is a tendinous arch formed by the proximal insertion of the abductor digiti minimi (ADM) and flexor digiti minimi brevis (FDM), whereas the superficial branch (arrowhead) keeps to its path superficial to the hypothenar muscles before branching in its terminal division. FR, flexor retinaculum; FCU, flexor carpi ulnaris; FT, flexor tendons; UA, ulnar artery; TM, thenar muscles. **b** Schematic drawing demonstrates the terminal divisions of the superficial (outlined arrowhead) and deep branch (arrowhead) distal to the Guyon tunnel. Thin arrow, proper ulnar palmar digital nerve for the V finger; dashed thin arrow, common palmar digital nerve for the IV webspace; #1 nerve for the hypothenar muscles, fourth lumbrical, fifth carpometacarpal and metacarpophalangeal joints; #2 nerve for the interossei of the third space; #3 nerve for the oblique head of the adductor pollicis; #4 nerve for the interossei of the fourth space and the fourth metacarpophalangeal joint; #5 nerve for the third lumbrical and the fourth carpometacarpal joint; #6 nerve for the interossei of the first and second spaces, the first and second intermetacarpal and metacarpophalangeal joints, the two heads of the adductor pollicis, and the deep head of the flexor pollicis brevis

The Guyon canal is a passageway through which the UN enters into the wrist and has been subdivided into three zones [4]. Zone #1 contains the main trunk of the UN and spans between the proximal edge of the palmar carpal ligament and the origin of the terminal branches of the UN. Nerve injuries in this area usually result in damage to both the sensory and the motor axons. Zone #2 corresponds to the path of the DB and spans from the end of Zone #1 to the level of the hamate hook. In detail, after branching from the UN distal to the pisiform, the DB dives around the ulnar slope of the hook traveling through the pisohamate hiatus, which is an opening in the tendinous arcade of the origin of the flexor digiti minimi brevis and abductor digiti minimi and represents the end of Zone #2 [5]. Nerve injuries in Zone #2 result in selective involvement of the motor axons, with possible sparing of the abductor digiti minimi in cases where the damage occurs distal to the origin of its supplying ramus. Distal to the hiatus, the DB pierces the opponens pollicis and arches in the palm traveling from ulnar to radial between the ventral interossei and the flexor tendons, pointing toward the thenar eminence. Along its path, the DB supplies the opponens digiti minimi, the III and IV lumbricals, the dorsal and ventral interossei, the adductor pollicis, and, in some instances, the opponens pollicis and the deep belly of the flexor pollicis brevis [6]. In cases of DB damage distal to Zone #2, the extension of muscle atrophy depends on the level of nerve injury, as if the DB is injured after the origin of the supplying branch for a specific muscle, this latter will be spared and will show normal tropism. Zone #3 corresponds to the path of the SB and its terminal branches and, contrary to Zone #1 and Zone #2, its distal border has not been clearly defined in anatomical descriptions. Considering its content, in around 80% of subjects the SB arises from the UN as a single trunk, whereas in the remaining cases, the two terminal divisions of the SB (e.g., the proper digital nerve for the ulnar side of the fifth finger and the common digital nerve of the fourth web space) arise directly from the main trunk of the UN, giving origin to a trifurcation of this latter [7]. In any case, the SB and its terminal divisions run at first beneath the palmaris brevis and superficial to the flexor digiti minimi muscles and then in the subcutaneous tissue of the palm [8] (Fig. 1).

## Materials and methods

Approval for this monocentric study, performed at the IRCCS Ospedale Policlinico San Martino (Genoa, Italy), was obtained from the competent Ethics Committee (Comitato Etico Regione Liguria, protocol code 12927, approved on 21/03/2023). We screened our institutional imaging database to identify patients with** a** US diagnosis of UN mononeuropathy performed in the five-year period between January 2018 and January 2023 and, among them, we reviewed the cases in which US identified pathological findings affecting the terminal divisions of the nerve. In this latter subgroup, we retrieved available data on MRI and electrodiagnostic tests performed by the patients during the diagnostic work-up. The flowchart of patient’s selection is summed up in Supplemental Fig. 1. We excluded patients in which we were able to retrieve clinical, imaging, or electrophysiological data suggesting a diagnosis of polyneuropathy. Patients were evaluated utilizing two different US machines depending on their availability at the time of examination (iU22® platform, Philips Healthcare, Bothell, WA, USA and i800 Aplio® platform, Canon Medical Systems, Ōtawara, Japan). US systems were equipped with 17–5-MHz, 18–4-MHz, and 24–8-MHz linear transducers or a 22–8-MHz hockey-stick probe. MRI was performed on a 3-T unit equipped with a 64-channel head RF coil (Magnetom Prisma®, Siemens Erlangen, Germany).

### Ultrasound and MRI protocols

During US evaluation, patients were invited to sit down in front of the examiner with the arm extended on the table and oriented palm-up. The UN was first identified at the axilla and then followed down in a short axis toward the elbow. At the elbow, the nerve was evaluated by asking the patients to maximally intrarotate the arm to better expose the area of the epitrochlear groove. In this area, the UN cross-sectional area was calculated to disclose potential Cubital tunnel syndrome using a cut-off of 10 mm^2^ [9]. In doubtful cases when mild nerve swelling at the Cubital tunnel was considered insufficient to explain patients’ symptoms, the contralateral UN was measured at the same level for comparison. At the Guyon tunnel, the UN was recognized on the radial side of the pisiform and was followed in a short axis across Zone #1 until its arborization. The evaluation of the SB was preferentially performed with the hockey-stick probe to better demonstrate its branching pattern from the UN and the path of its terminal divisions along the palm and the fingers. Linear probes were preferred to evaluate the DB at the pisohamate hiatus, around the hamate hook, and along its course underneath the flexor tendons. A careful scanning technique with transducer rotation along the axis, heel-toeing, and tilting was necessary to enhance the visibility of the DB. At the level of the hamate, the probe was tilted laterally to redirect the US beam perpendicular to the axis of the hook and to better depict the passage of the DB across the hiatus and the opponens digiti minimi. Distal to the opponens and across the palm, the probe was progressively rotated to image the DB in a short axis along its path underneath the flexor tendons. MRI investigation of the wrist and hand region was performed by placing the patient in the “superman” position with the hand placed at the isocenter, oriented palm down. The scanning protocol and sequence parameters are detailed in Table 1.

**Table 1:** MRI protocol and sequence parameters

	**FOV READ (mm)**	**FOV PHASE (mm)**	**TR (ms)**	**TE (ms)**	**VOXEL (mm)**	**Average**	**TA (min)**
tSE T1 coronal	150	100	442	15	0.3 × 0.3 × 2.5	3	3.1
tSE PD fs coronal	150	100	1840	36	0.3 × 0.3 × 2.5	4	3.15
tSE T2 fs axial	150	100	4650	97	0.3 × 0.3 × 3	5	4.25
tSE T1 axial	150	100	580	12	0.3 × 0.3 × 3	2	2.39
tSE PD fs sagittal	130	90.6	1600	36	0.3 × 0.3 × 2	4	6.35
3D tSE T2 fs sagittal	170	100	1000	37	0.7 × 0.7 × 0.7	1.6	6.52
tSE T2 axial HR (optional)	150	100	3250	97	0.3 × 0.3 × 2	7	5.45

## Results

### Demographics and imaging evaluation

A total of 166 patients with UN mononeuropathy (102 males, mean age = 55 years) were included in the study, and, among them, we identified fifteen (9%; 7 males, mean age = 57 years) cases with SB/DB damage. Seven patients had selective damage of the DB, four patients of the SB, and four patients had a combined neuropathy of the SB and DB. Thirteen (86.6%) patients with SB/DB damage had already been examined with electrodiagnostic tests before US. Seven (46.7%) patients were submitted to MRI to integrate US results. However, in only three (20%) cases, the additional information provided by MRI was judged of some utility in the following work-up of patients. In general, MRI was requested with the following purposes: (i) to characterize solid soft tissue masses; (ii) to identify the origin of ganglia when not possible with US; (iii) to obtain a panoramic view of the regional anatomy and to investigate ancillary findings such as carpal bone fractures, extensive soft tissue derangement, and vessels damage. In all patients, imaging findings significantly impacted therapeutic decisions, particularly guiding the choice between conservative and surgical treatment.

### Pathological findings

US identified seven traumatic nerve injuries, two nerve tumors, and six entrapment neuropathies, including four cases of nerve compression by a ganglion cyst.

#### DB neuropathies — entrapment syndromes

In six cases, US disclosed an entrapment neuropathy of the DB after its origin from the UN. Fusiform swelling proximal to the site of entrapment and abrupt thinning at the entrapment point represented the hallmarks of US diagnosis. Overall, three levels of potential entrapment of the DB were identified in our series, respectively consisting of (I) proximal to the pisohamate hiatus; (II) from the pisohamate hiatus to the opponens digiti minimi; and (III) from the opponens digiti minimi to the thenar eminence.

**Fig. 2:**
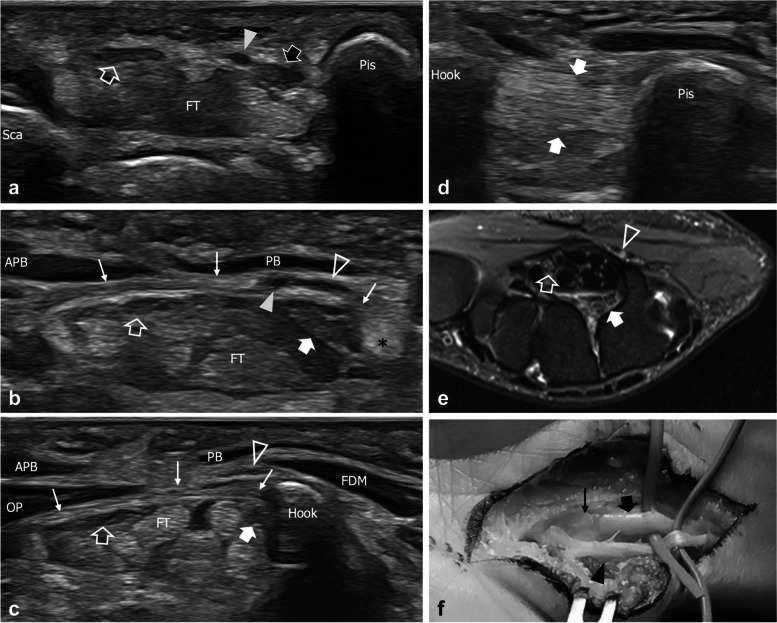
Level one entrapment neuropathy in a 29-year-old woman with progressive wasting of the interossei. **a**, **b**, **c** Consecutive short-axis 22–8-MHz US images from proximal to distal demonstrate a hypoechoic and swollen ulnar nerve (black arrow) at the proximal part of the Guyon canal, on the radial side of the pisiform (Pis). Distal to this level, the deep branch (white arrow) appears running in an anomalous position inside the carpal tunnel underneath the flexor retinaculum (thin arrows), on the radial side of a hypertrophied pisohamate ligament (asterisk) and, more distal, of the hamate hook, whereas the superficial branch (outlined arrowhead) keeps on running along the regular path on the side of the ulnar artery (arrowhead). Note the edematous changes and the swollen appearance of the deep branch compared to the regular median nerve (outlined arrow). Sca, scaphoid; FT, flexor tendons; APB, abductor pollicis brevis; PB, palmaris brevis; OP, opponens pollicis; FDM, flexor digiti minimi. **d** Long axis 22-8MHz US image better demonstrates the thickened pisohamate ligament (arrow). **e** Transverse turbo Spin Echo T2-weighted MRI scan with fat saturation shows the anomalous position of the deep branch (arrow) inside the carpal tunnel on the radial side of the hamate hook. The superficial branch (arrowhead) is normally positioned over the superficial aspect of the carpal ligament. Outlined arrow, median nerve. **f** The intraoperative picture confirms the swollen appearance of the deep branch (black arrow) before it engages the carpal tunnel underneath the flexor retinaculum (thin arrow). Arrowhead, superficial branch

**Fig. 3:**
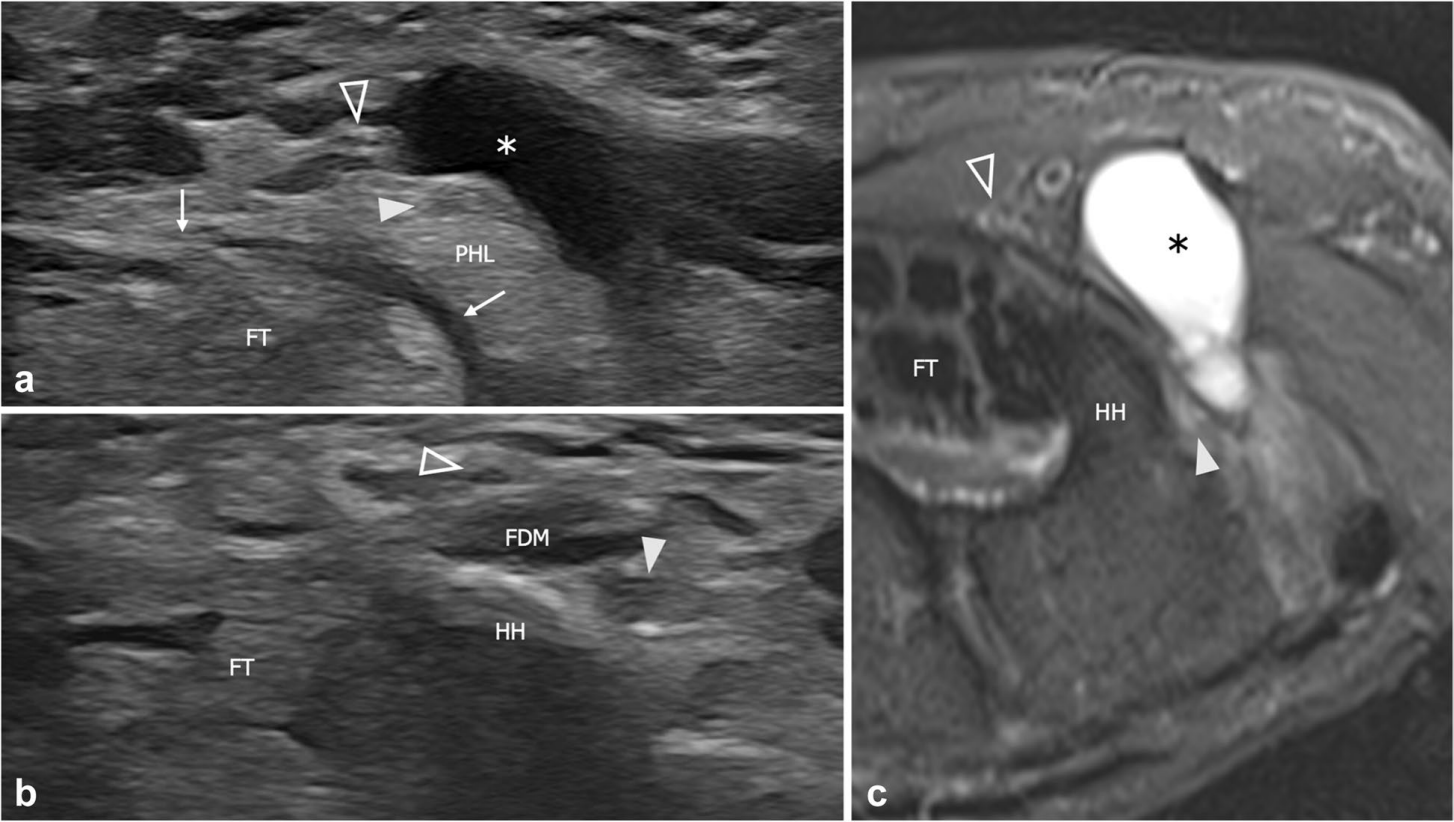
Level two extrinsic compression of the deep branch in a 61-year-old man reporting impairment in performing fine movements with the right hand. **a**, **b** Consecutive short-axis 18–5-MHz US images and **c** Transverse turbo Spin Echo T2-weighted MRI scan with fat saturation demonstrate a ganglion cyst (asterisk) that squeezes the deep branch (arrowhead) against the pisohamate ligament (PHL) and the hamate hook (HH). Note mild edematous changes with nerve swelling in **b**. The superficial branch (outlined arrowhead) is unaffected by the cyst. FT, flexor tendons; thin arrow, flexor retinaculum

In one patient, US disclosed an anomalous course of the DB inside the carpal tunnel with consequent nerve impingement at level one against the flexor retinaculum, the pisohamate ligament, and the hook of the hamate (Fig. 2). MRI was requested after US with the purpose of confirming the anomalous position of the DB inside the carpal tunnel, but the information added by this modality did not impact the following patient management. Three patients had a DB compression at level two by ganglion cysts arising from the pisotriquetral joint. These ganglia are usually a consequence of pisotriquetral joint osteoarthritis or instability and may extend into the pisohamate hiatus where they may impinge against the DB (Fig. 3). One of these patients required further investigation with MRI to better depict the cyst pedicle. In two patients, an entrapment of the DB distal to the opponens digiti minimi (level three) was diagnosed. In the first case, the DB was found entrapped by a fibrous band, and as US was not able to directly demonstrate the fibrous band, MRI was requested in the attempt to directly demonstrate the cause of nerve impingement (Supplemental Fig. 2). However, the revision of images showed that MRI did not add significant information to the one provided by US. The other patient had a mid-palm ganglion arising from the third carpometacarpal joint that squeezed the DB against the flexor tendons. In this case, MRI data were considered useful for surgical planning, as they provided a better depiction of the position of the ganglion and of its pedicle.

#### DB neuropathies — traumatic injuries

**Fig. 4:**
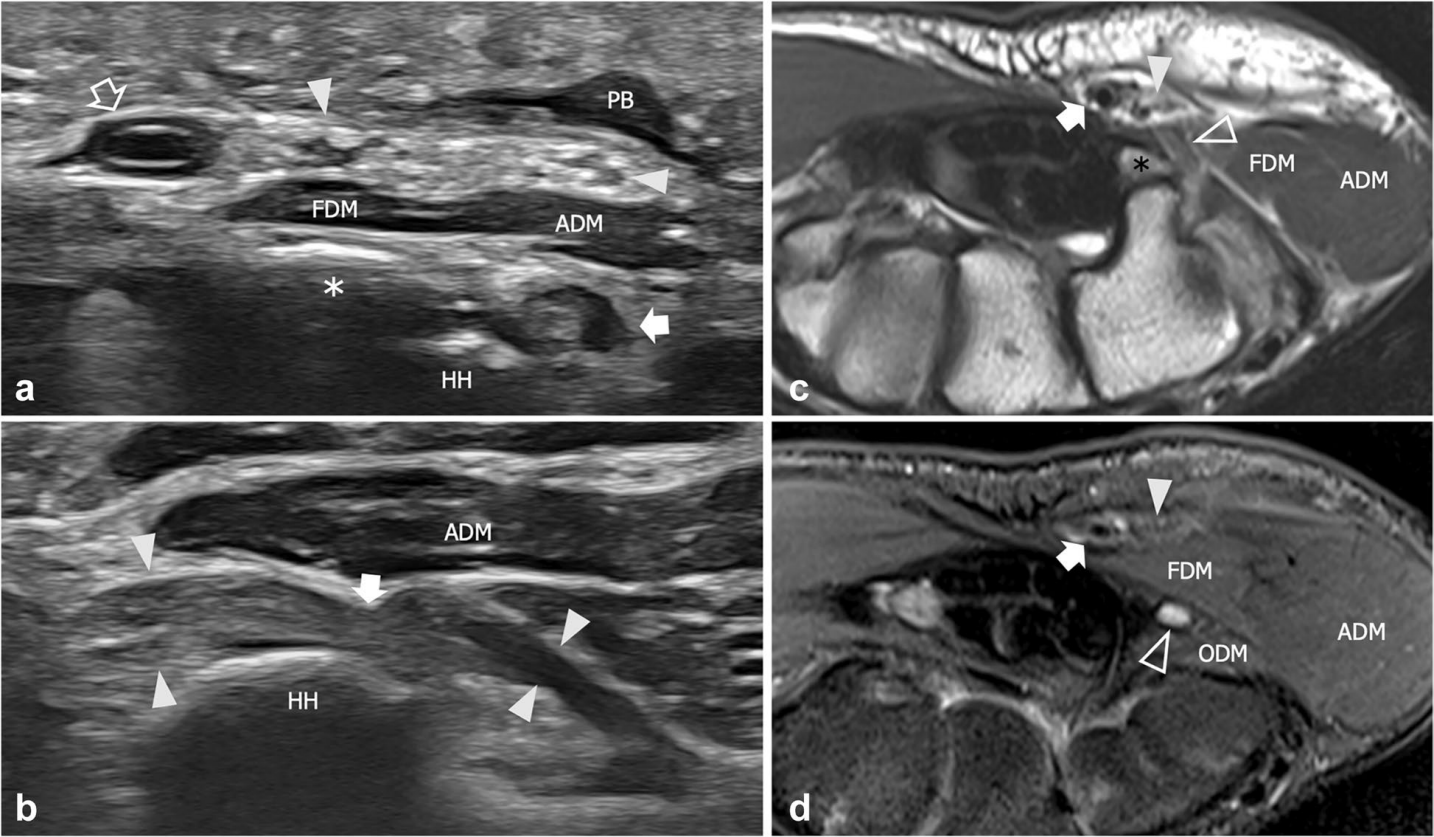
Deep branch impingement against an unrecognized hamate hook fracture in a 40-year-old male with motor impairment in the territory of the ulnar nerve after a blunt trauma over the palm. **a** Short-axis 22–8-MHz US image demonstrates severe edematous changes and swelling of the deep branch (arrow) as it runs on the ulnar side of the hamate hook (HH). A small piece of fractured bone (asterisk) is shown avulsed from the tip of the hook. The terminal divisions of the superficial branch (arrowheads) present a normal appearance as they run in between the palmaris brevis (PM) and the abductor digiti minimi (ADM) and flexor digiti minimi (FDM). Outlined arrow, ulnar artery. **b** Long axis 22-8MHz US image shows a swollen deep branch (arrowheads) presenting a distorted path around the level of the hamate hook. Note the abrupt change in caliper of the nerve at the hiatus (arrow), related to the impingement. **c** Transverse turbo Spin-Echo T1-weighted and **d** transverse turbo Spin-Echo fat suppressed T2-weighted MRI scans confirm swelling and edematous changes of the deep branch (outlined arrowhead) and the presence of a fracture of the hamate hook (asterisk). The ulnar artery (arrow) and the superficial branch (arrowhead) have a normal appearance

In one patient with a history of blunt trauma over the ulnar aspect of the palm and progressive motor impairment in the territory of the DB, US diagnosed a hamate hook fracture with DB impingement against the bone fragment (Fig. 4). Following US, MRI was requested with the aim of disclosing eventual associated injuries, but the information added by this modality did not impact the subsequent management.

#### SB neuropathies — traumatic injuries

High-resolution US was able to recognize a selective traumatic injury of the SB in three patients. The first had a complete transection of the SB following surgical release of the flexor retinaculum for carpal tunnel syndrome (Supplemental Fig. 3). In this case, MRI was requested after US, but this modality did not impact the following management. Two patients were diagnosed with overuse compression injury selectively involving the SB. This condition is encountered in patients with chronic trauma over the hypothenar eminence and has been included in the spectrum of the so-called cyclist handlebar palsy, which may involve one or both the terminal divisions of the UN. The detection on US of abnormal nerve enlargement in a patient with a supporting history was considered sufficient for diagnosis.

#### SB neuropathies — soft tissue tumors

In one patient, US allowed to demonstrate a millimetric lesion arising from the proper palmar digital nerve for the ulnar side of the little finger, a few millimeters distal to its origin from the SB (Supplemental Fig. 4). US findings allowed to hypothesize a diagnosis of schwannoma, which was confirmed at the histological exams through the identification of high levels of protein S100 expression in the lesion.

#### Combined neuropathies — traumatic injuries

**Fig. 5:**
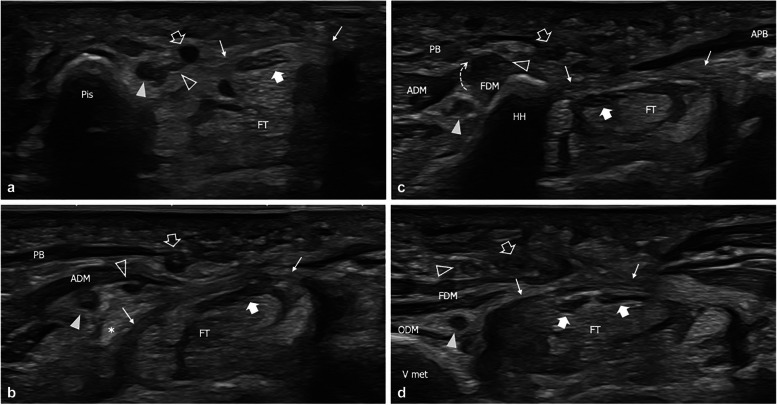
Compression neuropathy of the superficial and deep branches in a 62-year-old amateur cyclist with sensory numbness in the territory of the ulnar nerve. **a**, **b**, **c**, **d** Consecutive short-axis 18–5-MHz US images show mild edematous changes affecting the deep branch fascicles (arrowhead) at the level of the pisiform (Pis), with a normal appearance of the superficial branch (outlined arrowhead). In this area, the ulnar artery (outlined arrow) appears normal. **b** Between the pisiform and the hamate, the superficial branch has an anomalous path underneath the abductor digiti minimi (ADM). Note the thickening of the ulnar artery walls as it crosses the edematous subcutaneous tissue of the hypothenar eminence. **c** At the hamate hook (HH) the superficial branch crosses (dashed arrow) from deep to superficial the proximal part of the flexor digiti minimi (FDM). **d** At the base of the V metacarpus (V met) the deep branch presents a normal appearance as it runs between the flexor digiti minimi and the opponens digiti minimi (ODM) whereas the superficial branch appears swollen as it crosses the edematous subcutaneous tissues. Note the thrombosed ulnar artery on the radial side of the superficial branch. Arrow, median nerve; thin arrows, flexor retinaculum; APB, abductor pollicis brevis; Ft, flexor tendons; asterisk, pisohamate ligament

Three patients were diagnosed with traumatic injuries involving both the SB and the DB. One had a high-energy trauma on the volar aspect of the hand with transection of both the SB and the DB. Two other cases had an overuse injury over the hypothenar eminence with concomitant involvement of both the terminal divisions of the UN (Fig. 5). In these two cases of combined cyclist handlebar palsy, pathological findings affecting the DB were detected at the level of the hamate hook, as a consequence of the close anatomical relationship between the nerve and the underlying bone.

#### Combined neuropathies — soft tissue tumors

**Fig. 6:**
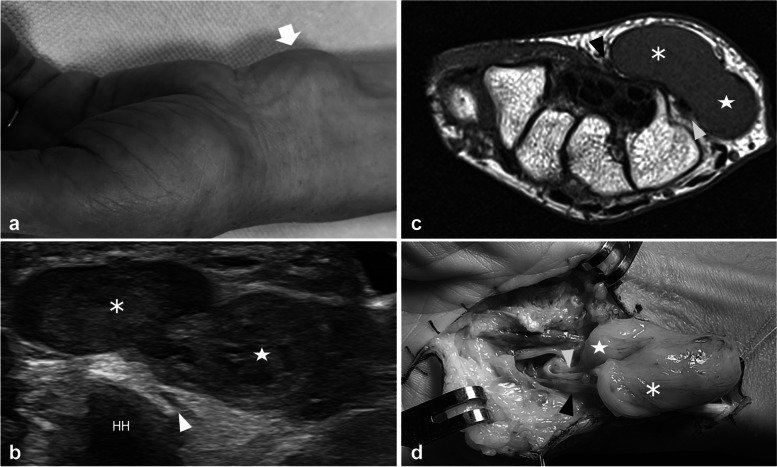
Ulnar nerve schwannoma involving the superficial and deep branch in a 54-year-old woman with an enlarging soft tissue mass on the ulnar side of the wrist. **a** The picture shows the appearance of the patient’s wrist at the time of US evaluation. **b** Short-axis 18-5MHz US image and **c** transverse turbo Spin-Echo T1-weighted MRI scan demonstrate a bilobate mass located in the soft tissue around the hamate hook (HH). The mass presents a deep and ulnar component (star) engaging the pisohamate hiatus and a superficial and radial component (asterisk) located superficial to the tip of the hook. Note the superficial branch fascicles (black arrowhead) displaced on the radial side of the superficial component of the mass and the deep branch (white arrowhead) compressed between the deep component of the lesion and the hook. **d** The intraoperative view shows the tumor arising from the main trunk of the ulnar nerve and extending distal with two distinct components into the superficial (black arrowheads) and the deep branches (white arrowhead). Histologic analysis confirmed a schwannoma

In one patient, US revealed a polylobate mass in continuity with the main trunk of the UN a few centimeters proximal to the Guyon tunnel (Fig. 6). At the distal Guyon, the lesion was demonstrated following the terminal division of the UN, with a deep part of the lesion engaging the pisohamate hiatus and growing around the DB and a superficial part expanding around the path of the SB. In this patient, MRI was requested for further characterizing the mass and to better demonstrate its anatomical location and its relationship with the adjacent structures. The integration of US and MRI data allowed to hypothesize a diagnosis of schwannoma, which was confirmed through histological analysis of the mass.

## Discussion

In our series, US allowed for the identification and characterization of SB/DB damage in 9% of patients with UN mononeuropathy. Interestingly, a significant 86.6% of patients with SB/DB damage had already been investigated with electrodiagnostic tests before imaging evaluation, but results were judged inconclusive, and further investigations were deemed necessary.

Electrodiagnostic tests currently stand as the first-line test for patients with suspected UN neuropathy at the wrist but hold several limitations in diagnosing SB/DB neuropathies [10, 11]. First, these modalities cannot differentiate between terminal division neuropathies and partial damage of the main nerve trunk with selective involvement of specific groups of fascicles at a more cranial level and may be misled by tandem lesions where SB or DB damage is superimposed to proximal UN neuropathy, for example in the case of compression at the cubital tunnel [12]. Additionally, electrodiagnostic tests do not provide any information about the underlying cause of neuropathy and may consequently prove unhelpful when deciding between conservative or surgical management. On the contrary, imaging modalities enable the direct demonstration of these submillimetric branches along their entire course and the precise identification of the cause of nerve damage. Previous works have already highlighted the efficacy of US and MRI in displaying the distal branches of the UN across the wrist and palm and in disclosing specific causes of neuropathy [13–15]. Furthermore, the greater accuracy of imaging modalities over electrodiagnostic tests in the evaluation of UN neuropathies has been previously suggested, although it is yet to be demonstrated in cohort studies [16].

In detail, in traumatic injuries around the wrist, imaging allows the differentiation between patients with neurapraxia and mild axonotmesis, who can be managed conservatively, and patients with severe axonotmesis and neurotmesis, who may require surgical intervention [17]. Conversely, electrodiagnostic tests have several limitations in defining the severity of the lesion, especially in the first weeks after trauma [18, 19]. Based on these considerations, US may be considered a first-line approach to subjects with suspected traumatic SB/DB injury, whereas electrodiagnostic tests could be reserved for cases where US findings are not conclusive. Meanwhile, in atraumatic SB/DB neuropathies, US can demonstrate extrinsic nerve compressions by space-occupying lesions, recognize anatomical aberrations leading to nerve impingement, and identify nerve tumors [20, 21]. Additionally, this modality presents significantly lower invasiveness and costs compared to electrodiagnostic tests. Following these considerations, it may be advisable to regard US as the first-line approach also in patients with suspected atraumatic SB/DB neuropathies. Nonetheless, further studies are necessary to demonstrate the superiority of one modality over the other.

In our study, the information provided by MRI was deemed to be of some utility for the subsequent patient management in only 20% of patients with a previous US diagnosis of SB/DB, suggesting that in a majority of cases, US results may be adequate for developing a therapeutic plan. Even if earlier works suggested a greater potential of US compared to MRI in revealing peripheral nerve pathology [22–24], there is presently a lack of evidence supporting the use of one modality over the other in these conditions. However, US is more available, has superior spatial resolution, and incurs lower costs. Based on these considerations and our experience, US may be regarded as a first-line approach in the context of imaging evaluation of peripheral neuropathies affecting the distal divisions of the UN, while MRI may be reserved for selected cases where US findings are deemed inconclusive. Generally, MRI offers some advantages in providing a panoramic view of large masses and may furnish additional information regarding their internal structure and their anatomical relationship with locoregional structures. Additionally, MRI may assist in tracking deeply located ganglia pedicles and provide complementary data regarding the status of the joint from which the ganglia arise [25].

Nevertheless, the present work does exhibit some limitations. Firstly, this is a retrospective study, and patients were not randomized to be initially evaluated with imaging modalities or electrodiagnostic tests, but were collected as a heterogeneous series of patients who were diagnosed with UN neuropathy by means of US. Although the goal of the present study was not to demonstrate the superiority of imaging modalities over electrodiagnostic tests, we intended to illustrate the potential of US and MRI in disclosing pathological findings in patients with SB/DB neuropathy. We acknowledge that US and MRI examination of terminal nerve branches requires high-end equipment and trained operators, and our results may not be generalizable to other centers. In addition, US and MRI examinations were performed by three different operators, and we do not have any data about the intra- and interobserver reliabilities of imaging modalities in the evaluation of the distal branches of the UN. Overall, the diagnostic work-up of SB/DB neuropathy should be tailored based on physical examination, anamnestic records, and local expertise.

In conclusion, high-resolution US and MRI are accurate modalities for the investigation of patients with SB/DB neuropathy. They may provide critical information on the cause of nerve damage and guide the subsequent management. In the proper setting, US may be regarded as a first-line approach in patients with suspected neuropathies affecting these small branches.

### Supplementary Information

**Additional file 1: Supplemental Fig. 1.** Flowchart of patients' selection. **Supplemental Fig. 2.** Deep branch entrapment by a fibrous band in a 45-year-old patient with progressive atrophy of the interossei of the first, second, and third webspace. (A) Oblique 17-5 MHz US image demonstrates a thickened deep branch (arrows) abruptly shrinking (arrowheads) along its path underneath the flexor digiti minimi (FDM) because of nerve constriction by a fibrous band. (B) Transverse 17-5 MHz US image obtained at the midshaft of the metacarpi shows atrophic changes affecting the interossei of the III webspace, whereas the muscles of the IV webspace are unaffected. **Supplemental Fig. 3.** Iatrogenic superficial branch injury in a 69-year-old female patient with pain and paresthesia in the territory of the ulnar nerve after surgical release of the flexor retinaculum for carpal tunnel syndrome. (A) Short-axis 18-5 MHz US image shows a wide scar (outlined arrows) along the surgical access reaching the superficial branch (arrowhead) a few millimeters after its origin from the ulnar nerve. The deep branch (outlined arrowhead) is unaffected by the scar, whereas the ulnar artery is not recognizable at this level. Note the swollen median nerve (arrow) underneath the flexor retinaculum (thin arrows). (B) Short-axis 18-5 MHz US image obtained at a more distal level than (A) demonstrates two terminal neuromas (arrowheads) affecting the divisions of the superficial branch as they run between the palmaris brevis (PB) and the flexor digiti minimi (FDM). The deep branch (outlined arrowhead) has a normal appearance on the ulnar side of the hamate hook (HH). Note the thrombosed ulnar artery (arrow). ADM, abductor digiti minimi. (C) Long-axis 18-5 MHz image shows the superficial branch (arrowheads) terminating in the post-surgical scar tissue (arrows). (D) Transverse turbo Spin Echo T1-weighted MRI scan confirms the transection of the superficial branch (arrowhead), which appears dispersed inside fibrotic tissue, and the regular appearance of the deep branch (outlined arrowhead). (E) Time-of-flight MRI demonstrates the absence of blood flow inside the transected and thrombosed ulnar artery in the hand. **Supplemental Fig. 4.** Schwannoma of the proper palmar digital nerve for the ulnar side of the V finger in a 43-year-old female patient with recent onset of tingling and paresthesia of the little finger. (A) Short-axis 22-8 MHz US image demonstrates the common palmar digital nerve for the IV space (arrowhead), the proper palmar digital nerve for the ulnar side of the V finger (outlined arrowhead), the deep branch (arrow) and the ulnar artery (outlined arrow) at the level of the hamate hook (HH). (B) Short-axis 22-8 MHz US image obtained a few millimeters distal to (A) shows a small schwannoma (outlined arrowhead) arising from the proper palmar digital nerve for the ulnar side of the V finger. Compare the appearance of the schwannoma with the normal-appearing proper palmar digital nerve for the ulnar side of the ring finger (black arrowhead) and the radial side of the little finger (void black arrowhead). PB, palmaris brevis; ADM, abductor digiti minimi; FDM, flexor digiti minimi brevis.

## Data Availability

The datasets used and/or analyzed during the current study are available from the corresponding author on reasonable request.

## Abbreviations

DB: Deep branch of the ulnar nerve
SB: Superficial branch of the ulnar nerve
UN: Ulnar nerve

## References

[1] Saracco M, Panzera RM, Merico B (2021). Isolated compression of the ulnar motor branch due to carpal joint ganglia: clinical series, surgical technique and postoperative outcomes. Eur J Orthop Surg Traumatol.

[2] Shea JD, McClain EJ (1969). Ulnar-nerve compression syndromes at and below the wrist. J Bone Jt Surg Am.

[3] Uriburu IJ, Morchio FJ, Marin JC (1976). Compression syndrome of the deep motor branch of the ulnar nerve. (Piso-Hamate Hiatus syndrome). J. Bone Joint Surg. Am..

[4] Gross MS, Gelberman RH (1985). The anatomy of the distal ulnar tunnel. Clin Orthop Relat Res.

[5] Maroukis BL, Ogawa T, Rehim SA (2015). Guyon canal: the evolution of clinical anatomy. J Hand Surg Am.

[6] Atkins SE, Logan B, McGrouther DA (2009). The deep (motor) branch of the ulnar nerve: a detailed examination of its course and the clinical significance of its damage. J Hand Surg Eur.

[7] Dępkat P, Henry BM, Popieluszko P (2017). Anatomical variability and histological structure of the ulnar nerve in the Guyon’s canal. Arch Orthop Trauma Surg.

[8] Sulaiman S, Soames R, Lamb C (2015). Ulnar nerve cutaneous distribution in the palm: application to surgery of the hand. Clin Anat.

[9] Fodor D, Rodriguez-Garcia SC, Cantisani V (2022). The EFSUMB Guidelines and Recommendations for Musculoskeletal Ultrasound - Part I: Extraarticular Pathologies. Ultraschall Med.

[10] Seror P (2013). Electrophysiological pattern of 53 cases of ulnar nerve lesion at the wrist. Neurophysiol Clin.

[11] Raeissadat SA, Youseffam P, Bagherzadeh L (2019). Electrodiagnostic Findings in 441 Patients with Ulnar Neuropathy - a Retrospective Study. Orthop Res Rev.

[12] Lee SU, Kim MW, Kim JM (2016). Ultrasound Diagnosis of Double Crush Syndrome of the Ulnar Nerve by the Anconeus Epitrochlearis and a Ganglion. J Korean Neurosurg Soc.

[13] Niitsu M, Kokubo N, Nojima S (2010). Variations of the ulnar nerve in Guyon’s canal: in vivo demonstration using ultrasound and 3 T MRI. Acta Radiol.

[14] Karvelas KR, Walker FO (2019). Clinical and Ultrasonographic Features of Distal Ulnar Neuropathy: A Review. Front Neurol.

[15] Iyer VG (2021). Ultrasonography in Distal Ulnar Nerve Neuropathy: Findings in 33 Patients. J Clin Neurophysiol.

[16] Agarwal A, Chandra A, Jaipal U (2019). Imaging in the diagnosis of ulnar nerve pathologies—a neoteric approach. Insights Imaging.

[17] Padua L, Di Pasquale A, Liotta G (2013). Ultrasound as a useful tool in the diagnosis and management of traumatic nerve lesions. Clin Neurophysiol.

[18] Elshewi IE, Fatouh MM, Mohamed RNES (2023). Value of ultrasound assessment for traumatic nerve injury of the upper limb. J Ultrasound..

[19] Wijntjes J, Borchert A, van Alfen N (2020). Nerve Ultrasound in Traumatic and Iatrogenic Peripheral Nerve Injury. Diagnostics (Basel).

[20] Kwak KW, Kim MS, Chang CH (2011). Ulnar nerve compression in Guyon’s canal by ganglion cyst. J Korean Neurosurg Soc.

[21] Scarborough A, MacFarlane RJ, Mehta N (2020). Ulnar tunnel syndrome: pathoanatomy, clinical features and management. Br J Hosp Med (Lond).

[22] Goyal A, Wadgera N, Srivastava DN (2021). Imaging of traumatic peripheral nerve injuries. J Clin Orthop Trauma.

[23] Lee FC, Singh H, Nazarian LN (2011). High-resolution ultrasonography in the diagnosis and intraoperative management of peripheral nerve lesions. J Neurosurg.

[24] Zaidman CM, Seelig MJ, Baker JC (2013). Detection of peripheral nerve pathology: comparison of ultrasound and MRI. Neurology.

[25] Neto N, Nunnes P (2016). Spectrum of MRI features of ganglion and synovial cysts. Insights Imaging.

