# Supplementary material for: High-resolution ultrasound and magnetic resonance imaging of ulnar nerve neuropathy in the distal Guyon tunnel
Source: Insights Imaging. 2023 Nov 28;14:210. doi: 10.1186/s13244-023-01545-z (PMC10684459; doi:10.1186/s13244-023-01545-z)
Supplement: Supplementary file 1 — Additional file 1: Supplemental Fig. 1. Flowchart of patients' selection. Supplemental Fig. 2. Deep branch entrapment by a fibrous band in a 45-year-old patient with progressive atrophy of the interossei of the first, second, and third webspace. (A) Oblique 17-5 MHz US image demonstrates a thickened deep branch (arrows) abruptly shrinking (arrowheads) along its path underneath the flexor digiti minimi (FDM) because of nerve constriction by a fibrous band. (B) Transverse 17-5 MHz US image obtained at the midshaft of the metacarpi shows atrophic changes affecting the interossei of the III webspace, whereas the muscles of the IV webspace are unaffected. Supplemental Fig. 3. Iatrogenic superficial branch injury in a 69-year-old female patient with pain and paresthesia in the territory of the ulnar nerve after surgical release of the flexor retinaculum for carpal tunnel syndrome. (A) Short-axis 18-5 MHz US image shows a wide scar (outlined arrows) along the surgical access reaching the superficial branch (arrowhead) a few millimeters after its origin from the ulnar nerve. The deep branch (outlined arrowhead) is unaffected by the scar, whereas the ulnar artery is not recognizable at this level. Note the swollen median nerve (arrow) underneath the flexor retinaculum (thin arrows). (B) Short-axis 18-5 MHz US image obtained at a more distal level than (A) demonstrates two terminal neuromas (arrowheads) affecting the divisions of the superficial branch as they run between the palmaris brevis (PB) and the flexor digiti minimi (FDM). The deep branch (outlined arrowhead) has a normal appearance on the ulnar side of the hamate hook (HH). Note the thrombosed ulnar artery (arrow). ADM, abductor digiti minimi. (C) Long-axis 18-5 MHz image shows the superficial branch (arrowheads) terminating in the post-surgical scar tissue (arrows). (D) Transverse turbo Spin Echo T1-weighted MRI scan confirms the transection of the superficial branch (arrowhead), which appears dispersed inside fibr [file 13244_2023_1545_MOESM1_ESM.docx]

**High-resolution Ultrasound and Magnetic Resonance Imaging of Ulnar Nerve Neuropathy in the distal Guyon Tunnel**

**ELECTRONIC SUPPLEMENTARY MATERIAL**

**Supplemental Fig. 1:** Flowchart of patients' selection


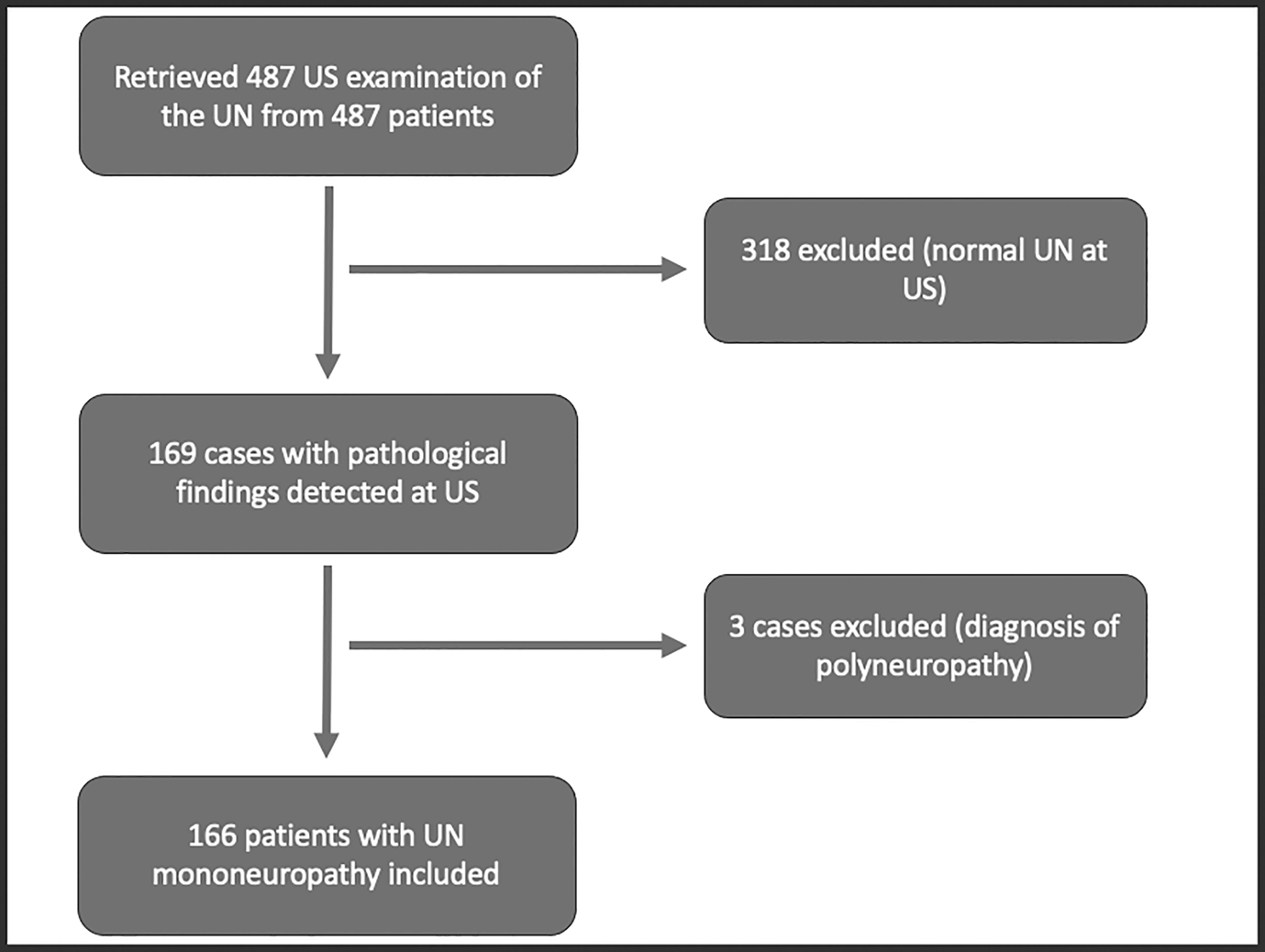


**
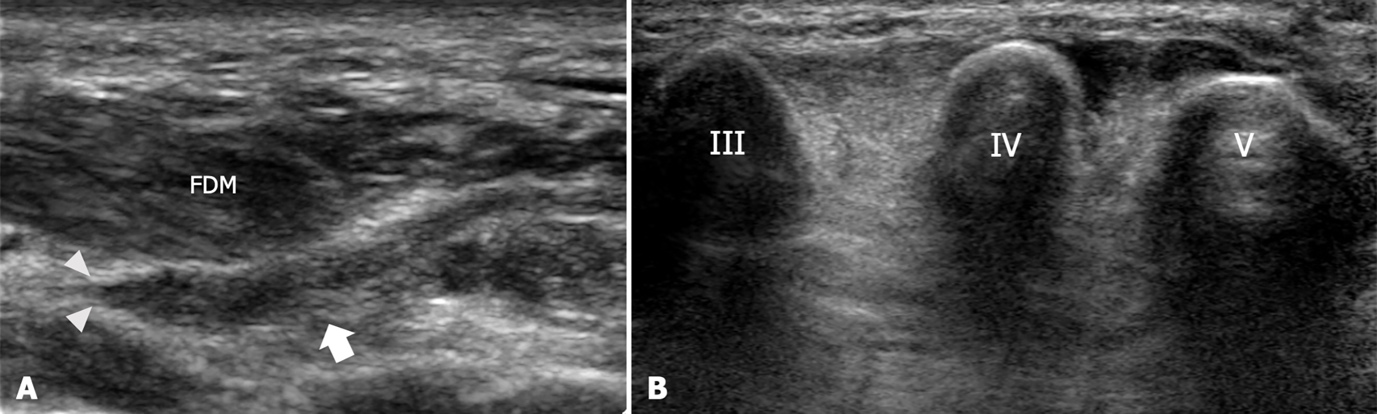
**

**Supplemental Fig. 2:** Deep branch entrapment by a fibrous band in a 45-year-old patient with progressive atrophy of the interossei of the first, second, and third webspace. (A) Oblique 17-5MHz US image demonstrates a thickened deep branch (arrows) abruptly shrinking (arrowheads) along its path underneath the flexor digiti minimi (FDM) because of nerve constriction by a fibrous band. (B) Transverse 17-5MHz US image obtained at the midshaft of the metacarpi shows atrophic changes affecting the interossei of the III webspace, whereas the **muscles** of the IV webspace are unaffected.


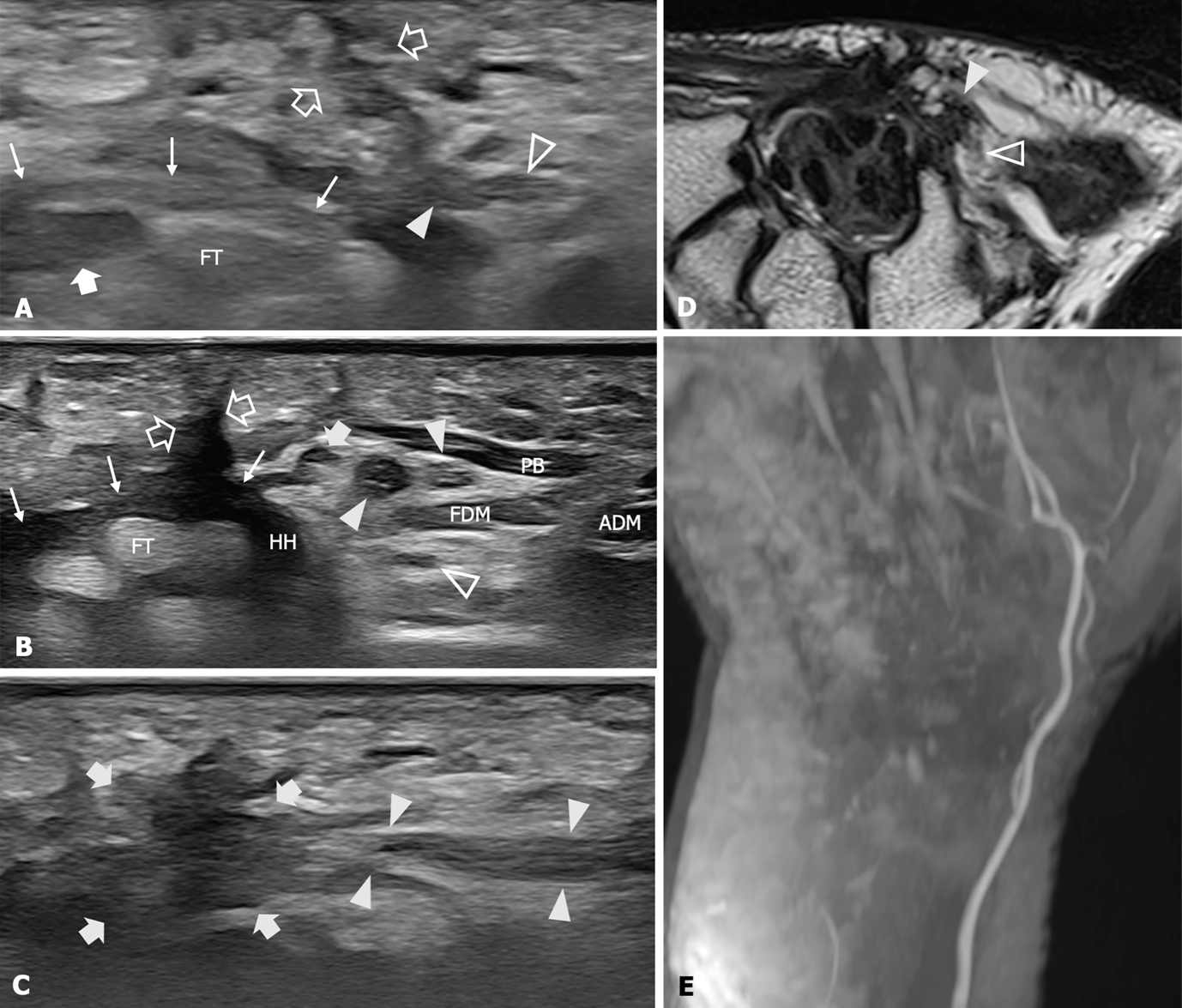


**Supplemental Fig. 3:** Iatrogenic superficial branch injury in a 69-year-old female patient with pain and paresthesia in the territory of the ulnar nerve after surgical release of the flexor retinaculum for carpal tunnel syndrome. (A) Short-axis 18-5MHz US image shows a wide scar (outlined arrows) along the surgical access reaching the superficial branch (arrowhead) a few millimeters after its origin from the ulnar nerve. The deep branch (outlined arrowhead) is unaffected by the scar, whereas the ulnar artery is not recognizable at this level. Note the swollen median nerve (arrow) underneath the flexor retinaculum (thin arrows). (B) Short-axis 18-5MHz US image obtained at a more distal level than (A) demonstrates two terminal neuromas (arrowheads) affecting the divisions of the superficial branch as they run between the palmaris brevis (PB) and the flexor digiti minimi (FDM). The deep branch (outlined arrowhead) has a normal appearance on the ulnar side of the hamate hook (HH). **Note** the thrombosed ulnar artery (arrow). ADM, abductor digiti minimi. (C) Long-axis 18-5MHz image shows the superficial branch (arrowheads) terminating in the post-surgical scar tissue (arrows). (D) Transverse turbo Spin Echo T1-weighted MRI scan confirms the transection of the superficial branch (arrowhead), which appears dispersed inside fibrotic tissue, and the regular appearance of the deep branch (outlined arrowhead). (E) Time-of-flight MRI demonstrates the absence of blood flow inside the transected and thrombosed ulnar artery in the hand.


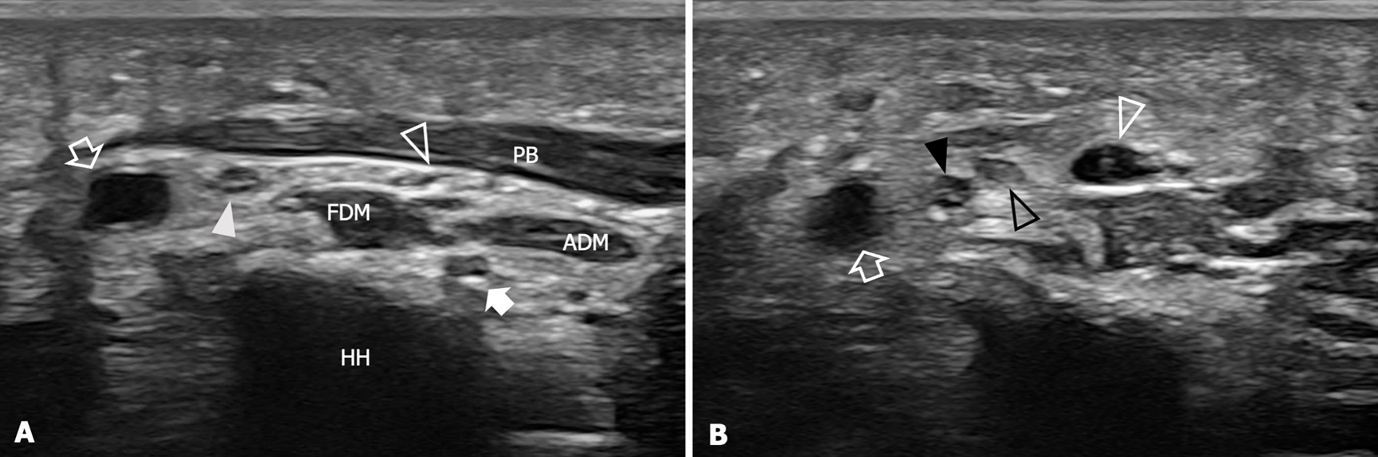


**Supplemental Fig. 4:** Schwannoma of the proper palmar digital nerve for the ulnar side of the V finger in a 43-year-old female patient with recent onset of tingling and paresthesia of the little finger. (A) Short-axis 22-8MHz US image demonstrates the common palmar interdigital nerve for the IV space (arrowhead), the proper palmar digital nerve for the ulnar side of the V finger (outlined arrowhead), the deep branch (arrow) and the ulnar artery (outlined arrow) at the level of the hamate hook (HH). (B) Short-axis 22-8MHz US image obtained a few millimeters distal to (A) shows a small schwannoma (outlined arrowhead) arising from the proper palmar digital nerve for the ulnar side of the V finger. Compare the appearance of the schwannoma with the normal-appearing proper palmar digital nerve for the ulnar side of the ring finger (black arrowhead) and the radial side of the little finger (void black arrowhead). PB, palmaris brevis; ADM, abductor digiti minimi; FDM, flexor digiti minimi brevis.
